# Effects of Furrow Irrigation on the Growth, Production, and Water Use Efficiency of Direct Sowing Rice

**DOI:** 10.1100/tsw.2010.146

**Published:** 2010-08-03

**Authors:** Chunlin He

**Affiliations:** ^1^ College of Agriculture, Guangdong Ocean University, Zhanjiang, China; ^2^ College of Life Science and Technology, Sichuan Agriculture University, Yaan, China

**Keywords:** conventional irrigation (CI), direct sowing rice, furrow irrigation (FI), water-saving mechanisms

## Abstract

Rice farming is the major crop production in Asia and is predicted to increase significantly in the near future in order to meet the demands for the increasing human population. Traditional irrigation methods used in rice farming often result in great water loss. New water-saving methods are urgently needed to reduce water consumption. Three field and pot experiments were conducted to evaluate the furrow irrigation (FI) system to improve water use efficiency (WUE) and production of direct sowing rice in southern China. Compared to the conventional irrigation (CI) system (continuous flooding irrigation), for every square hectometer of rice field, the FI system reduced water use by 3130 m^3^, or 48.1%, and increased grain production by 13.9% for an early cultivar. For a late cultivar, the FI system reduced water use by 2655 m^3^, or 40.6%, and an increase of grain production by 12.1%. The improved WUE in the FI system is attributed to (1) a significant reduction of irrigation rate, seepage, evaporation, and evapotranspiration; (2) a significant reduction in the reduced materials, such as ferrous ion (Fe^2+^), and therefore an increase in the vitality of the root system, evident by the increases in the number of white roots by 32.62%, and decreases in the number of black roots by 20.04% and yellow roots by 12.58%; the use of the FI system may also reduce humidity of the rice field and enhance gas transport in the soil and light penetration, which led to reduced rice diseases and increased leaf vitality; and (3) increases in tiller and effective spikes by 11.53% and the weight per thousand grains by 1.0 g. These findings suggest that the shallow FI system is a promising means for rice farming in areas with increasing water shortages.

### INTRODUCTION

Rice is the major human food in Asia, where about 92% of the world's rice is produced and consumed[1]. It is predicted that rice production has to be increased by 70% of the present amount by the year 2025 in order to meet the increasing demand[2]. More than 75% of the world's rice supply is produced in Asia using irrigated land that accounts for 50% of total freshwater use[3]. China is the most populated country and the largest rice producer in the world. The total area of rice farming is about 31 million ha and makes up 30% of the total planting area of grain crop. The annual rice production is approximately 185 million tons and accounts for 44% of the total grain production in China[4]. With rapid human population growth and industrial, urban, and agricultural development, water shortages have become a major problem in China as well as in other developing countries. On average, there are only about 2000 $m^3$ of freshwater per person per year in China, which is less than one-quarter of the world average. In addition, the rainfall pattern has become more variable in recent years as a result of global climate changes, with severe and unpredictable drought and flooding[4,5]. Tuong and Bouman[6] estimated that by 2025, 2 million ha of irrigated dry-season rice and 13 million ha of irrigated wet-season rice may experience water scarcity, and most of approximately 22 million ha of irrigated dry-season rice in Asia may suffer economic water scarcity.

Flooding irrigation is the traditional and most popular system for rice farming in Asia. As the problem associated with water shortages increases, there has been increasing research on effective water use in rice farming (e.g., [3,7,8,9]). Techniques such as controlled irrigation, intermittent irrigation, shallow irrigation, alternate wetting and drying (AWD), and low-density drought farming have been investigated[5,10,11,12]. Field water management to save water often leads to increased water productivity or water use efficiency (WUE) or slightly reduced rice yield[13]. Bouman and Tuong[14] reported that water saving under saturated soil conditions was on average 23% with yield reduction of only 6%. Yields were reduced by 10–40% when soil water potential in the root zone was allowed to reach –100 to –300 mbar. Water productivity in continuously flooded rice fields was typically 0.2–0.4 g grain per kg water in India and 0.3–1.1 g grain per kg water in the Philippines. Water-saving irrigation increased water productivity up to 1.9 g grain per kg water. Bouman et al.[15] reported that the water productivity of rice under aerobic conditions was 32–88% higher than under flooded conditions. Tao et al.[16] reported findings using a new water-saving Ground Cover Rice Production System (GCRPS), where lowland rice was cultivated without a standing water layer during the entire growth period. Compared to the control, only 32–54% of irrigation water was applied in the GCRPS treatments, but the yield was only 8% lower than the control. However, WUE was higher (0.35) than in the control (0.23). Feng et al.[11] indicated that by using AWD on lowland rice, irrigation water savings were 40–70% without any yield loss.

There are a number of studies that address morphological and physiological responses of rice plants to the changes in the irrigation regime. For example, Yang et al.[17] indicated that a moderate water deficit during the grain-filling period may induce early senescence that, however, enhances the remobilization of stored assimilates and accelerates grain filling of rice. Such enhancement may improve grain yield and also contribute to water saving in rice production. Lu et al.[8] compared dry mass and several physiological parameters of rice plants under continuous flooding and intermittent flooding irrigation conditions. They found no significant difference in WUE, dry mass, and leaf area index between the two irrigation methods. Tao et al.[16] found that rice in the GCRPS mentioned above was smaller, developed fewer panicles, and had a smaller leaf area index compared to control. The number of productive tillers had the greatest positive effect on yield, while the number of grains per panicle, thousand-grain weight, and harvest index remained almost unaffected. Nguyen et al.[12] indicated that the stomatal conductance was found to be most sensitive to a water reduction of 27–13% in a field study. The leaf elongation rate was also reduced.

One of the irrigation methods in agriculture is furrow irrigation (hereinafter referred to FI). Water from the delivery burrow laterally percolates into the vegetated bed. The absence of a surface water layer in the field reduces evaporation. This technique has been used for a variety of farmed plants[18,19,20,21]. Furthermore, the investigations on water-saving irrigation were largely based on farming of transplanted rice. There is an apparent lack of systematic studies on the methods and associated mechanisms of direct sowing rice farming and effective irrigation. Compared to transplanted rice, direct sowing may reduce water input by shortening the land precipitation period[3]. The objective of this study was to test the FI technique in a direct sowing rice field. Experiments on the growth and WUE of direct sowing rice were performed using the FI technique in the Leizhou Peninsula of southwestern Guangdong, China between 2002 and 2003, and provide a theoretical and empirical basis for the promotion of this technique at a large-scale rice field.

### MATERIALS AND METHODS

#### Study Sites and Experiment Designs

Three experiments were conducted at the Guangdong Ocean University Experimental Farm and Dongyang Research and Demonstration Station, both of which are located in the Leizhou Peninsula, a subtropical region in southwestern China, between 2002 and 2003. Experiments included field and pot simulation trials. The purpose of the field experiments was to measure WUE, rice growth parameters, and production with the FI and CI systems. The purpose of the pot experiment was to evaluate WUE under more controllable conditions.

The first field experiment was conducted at the Guangdong Ocean University Experimental Farm in 2002. The soil used in the trial was clay paddy soil from the top 0–20 cm with organic matter content of 16.8 g $kg^{-1}$, nitrogen content of 61.8 mg $kg^{-1}$, available phosphorus and potassium content of 2.9 mg $kg^{-1}$ and 30.6 mg $kg^{-1}$, respectively. Two infiltration irrigation rates with shallow furrow (FI-1 and FI-2) were used with the conventional irrigation (CI, i.e., continuous flooding) as control. The width of the rice bed was 200 cm, and the width and depth of the furrows were 20 cm and 15–18 cm, respectively. The total area of each plot was 166.68 $m^2$. Each experiment was run using the randomized complete block design with triplicate plots. Fertilization and field management were similar to those of the conventional rice farming in this region. Paddy field levees were covered with plastic film to prevent infiltration and leakage. The rice variety used in this experiment was a hybrid “Liang You Pei Jiu 65002”. Direct sowing was initiated on July 26, 2002 after soaking germination and harvesting took place on November 20, 2002.

A simulation pot trial was conducted simultaneously along with the field experiment at the Guangdong Ocean University Experimental Farm in 2002. Each polyethylene pot (diameter = 34 cm and height = 40 cm) was filled with 10 kg topsoil collected from the rice field. The trial contained six treatments, including leakage with water layer, leakage without water layer, evaporation with water layer, evaporation without water layer, evapotranspiration with water layer, and evapotranspiration without water layer. Each treatment had three replicates. Pot leakage for the treatments of leakage with and without water layer was estimated by collecting the seepage water from the bottom of each pot. Water consumption was measured during the trial. For the trial involved with evapotranspiration with and without water layer, evapotranspiration was estimated by the difference between total irrigation and evaporation. The trial was conducted under a shelter covered by a plastic film to prevent direct wet precipitation.

A second field experiment was conducted at the Dongyang Research and Demonstration Station in the Leizhou Peninsula in early 2003 to verify the results from the field and pot trial conducted at the Guangdong Ocean University Experimental Farm in 2002. This experiment contained two FI treatments and CI as control, with three replicates per treatment (total area: 0.8 $hm^2$). Seed germination took place on February 26, 2003 and the crop was harvested on July 1, 2003, with a total growth period of 126 days.

#### Measurements of Infiltration Rate and Water Budget

To determine soil water infiltration rate, a rectangular frame (length = 150 cm, width = 20 cm, and depth = 20 cm) imbedded with a glass sheet was placed in the field. The soil within the frame was evacuated. When the water content of the soil dropped to 75% of field capacity, water was allowed to flow into the irrigation furrow. Infiltration in the soil was directly observed and measured through the glass surface.

For all treatments using the FI technique at the field scale, a shallow water layer was maintained for 3–5 days during the time the dead seedlings were replaced. A brief period of rapid flow was initiated before fertilization. For the majority of the experimental period, irrigation was supplied via the irrigation furrow and there was no water layer in the experiment rice bed. In contrast, a shallow water depth was typically maintained for the control (CI).

Irrigation and discharge rates were measured with a flow meter and rainfall was measured with a rain gauge. Water use was estimated as the difference between total water input (irrigation + rainfall) and discharge[22]. Crop WUE, or yield per unit of water used, was calculated by dividing rice yield (kg $hm^{-2}$) by water use ($m^3$ $hm^{-2}$).

### Measurements of Biological Parameters

For the 2002 late rice experiment, when the rice seedlings reached the four-leave stage, each plot (50 × 50 cm) was planted with 28 seedlings. Seedling height and the number of tiller and green leaves in the main stem were recorded. At each of the growth stages, five rice plants from each plot were sampled and measured for length, number and diameter of roots, and the number of white, yellow, and black roots in the laboratory. Also at the maturity stage, a soil core (12 cm in diameter and 40 cm in depth), which included a rice plant above the soil surface and the underlying soil, was retrieved and sectioned at 10-cm intervals. Roots at each interval were isolated from the associated soil, washed-cleaned, and then measured for the weight and volume of roots.

Leaf area index (LAI) was calculated as 0.7575ΣLiWi[23] where Li and Wi are the length and width (mm) of the leaves, respectively. Canopy chlorophyll content was measured using a POC-1 chlorophyll meter. For the determination of dry weight, plant leaves, stems, and roots from randomly selected rice individuals were taken and measured for fresh weight, then dried at 105°C for 30 min and at 80°C for 24 h to attain constant weight. Content of ferrous iron ($Fe^{2+}$) in the soil was determined following standard method from the rice field on the 70th day of the field trial at the Dongyang Experimental Station in 2003.

### Data Analyses

Duncan’s multiple comparisons to assess significant differences between and among treatments were performed using SPSS statistical software (SPSS Inc., Chicago). Differences were considered significant at $p < 0.05$.

## RESULTS

### Water Infiltration Rate, Water Budget, and Water Use Efficiency

The movements of the waterfront after irrigation at different times showed that it took 15 min for the irrigation water to reach 52 cm of the rice bed and 30 min to reach the 115 cm of the rice bed. This indicates that it required less than 1 h to irrigate the entire rice bed with a width of 200 cm (Fig. 1).

Water use in the rice field consists of seepage, evaporation, and evapotranspiration. Infiltration of irrigation water by shallow furrow apparently reduced water use by reducing water use at all three aspects. The average water use for the conventional 2002 late rice was 6530 $m^3$ $hm^{-2}$ with a total grain production of 6530 kg $hm^{-2}$. This is compared to the average water use of 4033 $m^3$ $hm^{-2}$ and grain production of 6940 kg $hm^{-2}$ using the FI technique. This accounted for a 38.2% reduction of water use and a 6.4% increase in rice production (Table 1).

Results from the pot experiment indicated that compared to CI, the amount of evaporation, evapotranspiration, and seepage using the FI technique was reduced by 23.64, 29.63, and 74.15% with the total water saving of 37.84% (Fig. 2). Compared to the open field experiment, the water use in the pot experiment was considerably higher due to the absence of groundwater input, greater edge effect, and higher evaporation and evapotranspiration. WUE between the CI and FI treatment for both experiments was significantly different, with high WUE in FI than in CI (Table 1). WUE in FI-1 with higher irrigation rate was not different from that in FI-2 with lower irrigation rate.

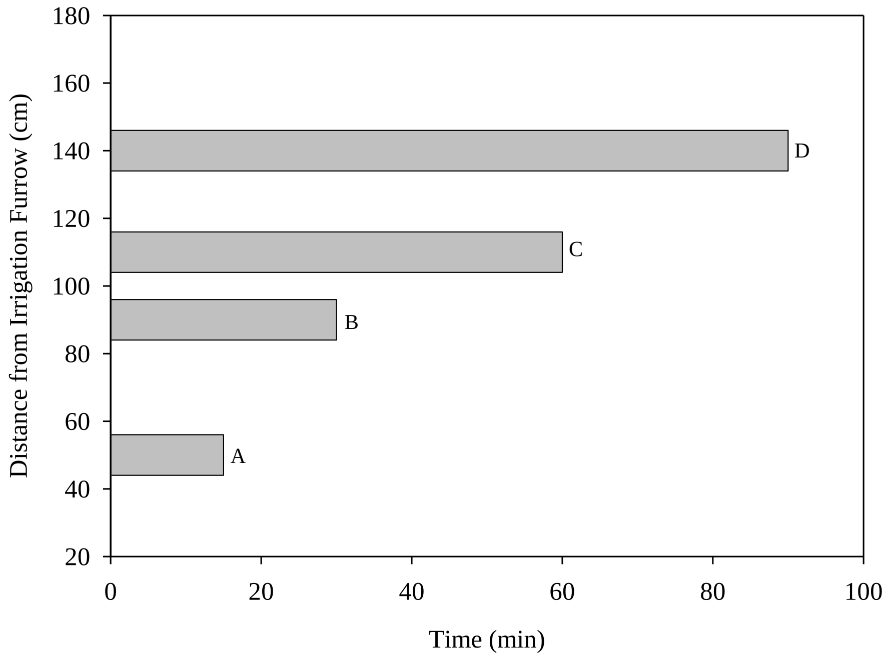

**FIGURE 1.** Permeating velocity of irrigated water at 20-cm depth of clay rice soil at different times: (A) 15 min, (B) 30 min, (C) 60 min, and (D) 90 min.

**TABLE 1**
**A Comparison of Water Use and Rice Production using the CI, FI-1, and FI-2 Treatments Conducted at Guandong Ocean University Experiment Station in 2002 and at Dongyang Research and Demonstration Station in the Leizhou Peninsula in early 2003**

| Year | Treatment | Irrigation ($m^3$ $hm^{-2}$) | Rainfall ($m^3$ $hm^{-2}$) | Discharge ($m^3$ $hm^{-2}$) | Water Use ($m^3$ $hm^{-2}$) | Water Saving ($m^3$ $hm^{-2}$) | Rice Yield (kg $hm^{-2}$) | WUE (kg $m^{-3}$ $hm^{-2}$) |
|---|---|---|---|---|---|---|---|---|
| 2002 | CI | 4384 | 6415 | 4268 | 6530 | 0 | 6530[aa] | 1.00[aa] |
| | FI-1 | 2464 | 6415 | 4846 | 4033 | 38 | 6940[bb] | 1.72[bb] |
| | FI-2 | 2290 | 6415 | 4988 | 3717 | 43 | 7090[bb] | 1.91[bb] |
| 2003 | CI | 5360 | 2106 | 966 | 6500 | 0 | 9525[a] | 1.46[a] |
| | FI | 2805 | 2106 | 1441 | 3370 | 48.1 | 10845[b] | 3.21[b] |

*Note*: Comparisons were made between treatments within year. Values with different letters are statistically different at $p < 0.05$.

### Effects of Irrigation Techniques on Biological Parameters of Rice

The average height for the direct sowing of the three growth stages in 2002 late rice was greatest for the CI, followed by FI-1 and FI-2. Plant height at FI-1 was 5.5, 2.4, and 3.4% lower than the CI at the three different growth stages, respectively. However, there were no differences in plant height among the three stages of FI-1 and FI-2. The number of tillers in all treatments decreased dramatically with growth, with more significant decline for the CI technique than for both FI treatments (Table 2).

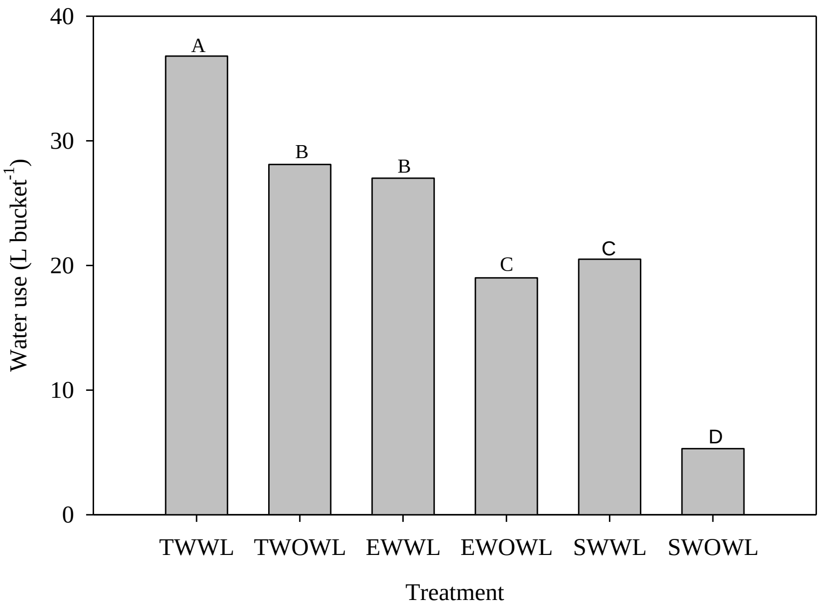

**FIGURE 2.** Effects of irrigation methods on water use in the pot simulation experiment. TWWL = transpiration with water layer; TWOWL = transpiration without water layer; EWWL = evaporation with water layer; EWOWL = evaporation without water layer; SWWL = seepage with water layer; SWOWL = seepage without water layer. Different letters at the top of the bars indicate significant difference ($p < 0.05$).

**TABLE 2**
**Plant Height and Tiller Numbers in the CI, FI-1, and FI-2 Treatments Conducted at Guandong Ocean University Experiment Station in 2002**

| Treatment | Plant Height (cm) | | | Tiller Number (0.25 $m^2$) | | |
|---|---|---|---|---|---|---|
| | Sept 8 | Oct 19 | Nov 9 | Sept 8 | Oct 19 | Nov 9 |
| CI | 26.1[a] | 75.3[a] | 78.9[a] | 225[a] | 114[a] | 101[a] |
| FI-1 | 24.7[b] | 73.5[b] | 76.2[b] | 211[b] | 118[a] | 106[a] |
| FI-2 | 24.1[b] | 73.0[b] | 75.6[b] | 206[b] | 110[a] | 102[a] |

*Note*: Values with different letters are statistically different at $p < 0.05$.

Stems are main channels for nutrient transport and storage sites for starch. Large stems are helpful for nutrient storage and transport. Stem dry weight in the CI was slightly greater than those in FI-1 or FI-2 on October 19, 2002, but the differences were not significant. By November 9, the stem dry weight for both treatments was higher than the CI method, with significant difference ($p < 0.05$), indicating faster growth of rice grown with the FI technique than the CI technique (Fig. 3).

The positive effects of the FI technique were also evident from the root system. The length and diameter of roots from the FI treatment were greater than those from the CI treatment at all stages of growth, with a general trend of FI-1 > FI-2 > CI (Duncan's multiple comparison, $p < 0.05$). At the tillering stage, these parameters were greater in FI-2 than FI-1; average root length was the greatest at the flowering stage. Differences among treatments were small at the tillering and the maturity stage, and

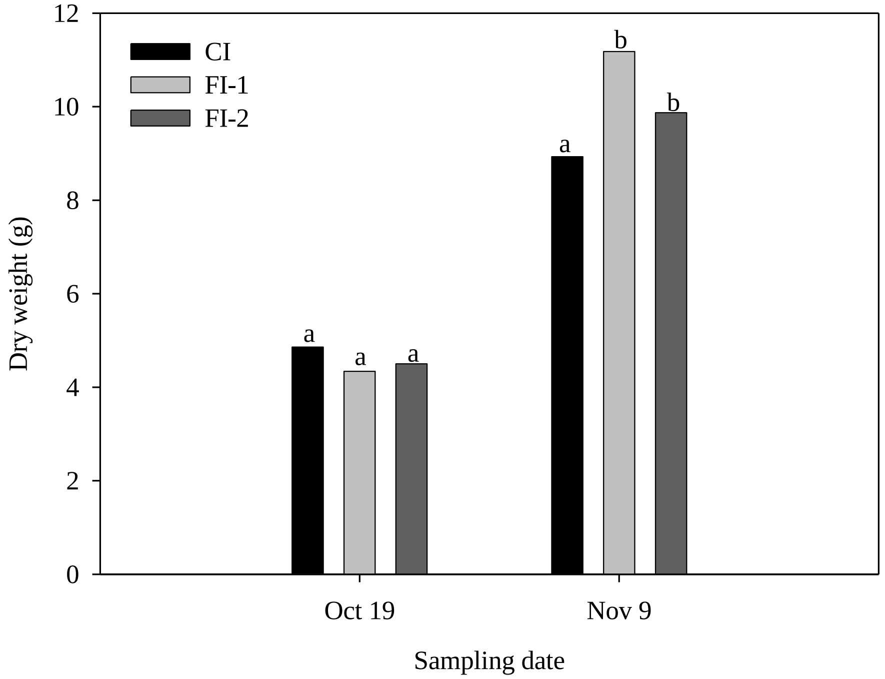

**FIGURE 3.** Comparisons of dry weight of stems among treatments at different sampling dates. Different letters at the top of the bars indicate significant difference ($p < 0.05$).

became greater at the flowering stage. At this stage, the average root length for FI-1 and FI-2 was 2.12 cm and 1.48 cm longer than that of CI. The average root diameter was greatest in FI-1, followed by FI-2 and CI. The total surface area of roots was positively associated with individual root length, indicating that there are greater numbers and surface area of the root system in the FI system than the CI system (Table 3).It was observed that 88–97% of the root biomass from the CI system was contained at the top 30 cm of soil. There were large variations in the distribution of the root biomass at a soil depth between 30 and 40 cm. Only about 3–4% of the roots were distributed at this layer in the CI system, while as many as 9–12% was found at this layer in the FI system (Table 4).

There were differences in root dry weight at different growth stages and in different treatments. Among treatments, root dry weight at all growth stages was greater in FI-2 than FI-1, followed by CI. At a temporal scale, there was a trend of an initial increase in root dry weight followed by a decline for all treatments, with significant differences among treatments. During the period from September 8 to October 19, root dry weight increased by 0.4 g in the CI treatment, 0.7 g in the FI-1, and 0.6 g in the FI-2. However, these differences were not significant. From the period of October 19 to November 9, root dry weight all decreased across treatments, with 0.9 g in the CI system, and 0.5 g in both the FI-1 and FI-2. The difference between CI and FI-1 and FI-2 was significant (Fig. 4).

Root color is an important indicator for root vitality. Results from the Dongyang Experiment Station in 2003 showed that yellow roots were the dominant roots (72.94%), followed by black (15.45%) and white roots (11.61%) in the CI system. Compared to the CI system, the yellow and black roots were reduced by 20.04 and 12.58% in the FI treatments, while the white roots increased by 32.62 and 44.23% of the roots in FI system (Table 5). This indicates that under the conventional flooding system, the root system of the paddy rice was deteriorated, likely due to the reduced conditions under long-term flooding that resulted in the depletion of oxygen. In contrast, under the permeable irrigation condition, gas conductance was high and the amount of reduced materials was low, allowing the development of a healthier root system.

**TABLE 3**
**Characteristics of the Root System in the CI, FI-1, and FI-2 Treatments Conducted at Guandong Ocean University Experiment Station in 2002**

| Parameter | Treatment | Growth Stage | | |
|---|---|---|---|---|
| | | Tillering | Flowering | Maturity |
| Total length (cm) | CI | 278[a] | 820[a] | 631[a] |
| | FI-1 | 382[b] | 1273[b] | 907[b] |
| | FI-2 | 371[b] | 1029[b] | 672[b] |
| Average length (cm) | CI | 5.15[a] | 6.77[a] | 6.17[a] |
| | FI-1 | 5.22[b] | 8.89[b] | 6.72[b] |
| | FI-2 | 5.40[c] | 8.25[b] | 6.57[b] |
| Average diameter (cm) | CI | 0.163[a] | 0.185[a] | 0.157[a] |
| | FI-1 | 0.178[b] | 0.225[b] | 0.178[b] |
| | FI-2 | 0.172[b] | 0.198[a] | 0.157[a] |
| Total area ($cm^2$) | CI | 142[a] | 476[a] | 311[a] |
| | FI-1 | 213[b] | 899[b] | 507[b] |
| | FI-2 | 200[c] | 640[c] | 331[a] |

*Note*: Comparisons were made among treatments for each parameter. Values with different letters are statistically different at $p < 0.05$.

**TABLE 4**
**Root Volume ($cm^3$) and Dry Weight (g) at Different Soil Depth in 2002 Late Rice Experiment Conducted at Guandong Ocean University Experiment Station**

| Soil Depth (cm) | 0–10 | | 10–20 | | 20–30 | | 30–40 | |
|---|---|---|---|---|---|---|---|---|
| Treatment | Volume | Weight | Volume | Weight | Volume | Weight | Volume | Weight |
| CI | 65[a] | 64[a] | 20[a] | 22[a] | 12[a] | 10[a] | 3[a] | 4[a] |
| FI-1 | 49[b] | 50[b] | 24[b] | 26[b] | 15[b] | 14[b] | 12[b] | 10[b] |
| FI-2 | 48[b] | 48[b] | 26[b] | 29[b] | 15[b] | 14[b] | 11[b] | 9[b] |

*Note*: Comparisons were made among treatments for each parameter and depth. Values with different letters are statistically different at $p < 0.05$.

The number of green leaves per plant is also affected by different irrigation methods. At the early tillering stage, the average number of green leaves per rice plant was 3.56 leaves for the CI system, 3.67 for FI-1, and 3.64 for FI-2. At the flowering and maturity stage, the number of green leaves and leaf area were also highest in FI-2, followed by FI-1 and CI (Table 6).

The difference in the length of the flag leaf was trivial between the two treatments ($p > 0.05$), but the difference ranged from 1.3 to 2.0 cm compared to those of the CI system. The difference in leaf width among treatments was small, but the difference in inclination (leaf angle) was significant, with a ranking from CI, FI-1, and FI-2**.** The length and orientation for the bottom second leaf and the third leaf in the CI system were greater than the FI treatments, indicating that the top third leaves were soft and spread, and the bottom leaves were weak, which may affect population photosynthesis (Table 7).

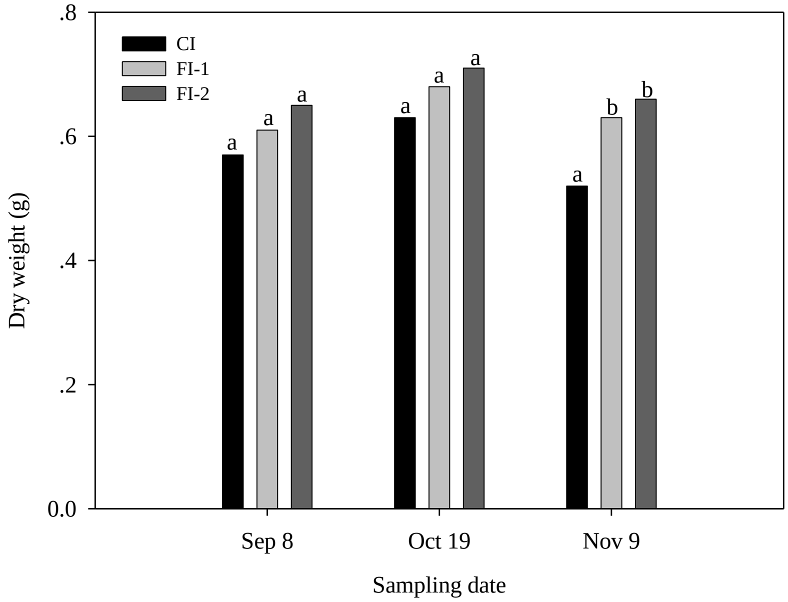

**FIGURE 4.** Comparisons of dry weight of roots among different treatments at different sampling dates. Different letters at the top of the bars indicate significant difference ($p < 0.05$).

**TABLE 5**
**Root Color and Soil Ferrous Iron Concentration in the CI and FI Treatments Conducted at Dongyang Research and Demonstration Station in the Leizhou Peninsula in Early 2003**

| Treatment | Total | White | | Yellow | | Black | | $Fe^{2+}$ (mg $kg^{-1}$) |
|---|---|---|---|---|---|---|---|---|
| | | Count | % | Count | % | Count | % | |
| CI | 179[a] | 20.8[a] | 11.61 | 130.7[a] | 72.94 | 27.7[a] | 15.45 | 133[a] |
| FI | 170[a] | 75[b] | 44.23 | 90.3[b] | 52.9 | 4.9[b] | 2.87 | 37[b] |

*Note:* Comparisons were made between treatments for each parameter. Values with different symbols or letters are statistically different at $p < 0.05$.

**TABLE 6**
**Number of Green Leaves and Leaf Area ($cm^2$) in the CI, FI-1, and FI-2 Treatments Conducted at Guandong Ocean University Experiment Station in 2002**

| Treatment | Tillering | | Flowering | | Maturity | |
|---|---|---|---|---|---|---|
| | # Green Leaves | Leaf Area | # Green Leaves | Leaf Area | # Green Leaves | Leaf Area |
| CI | 3.56[a] | 52.65[a] | 5.22[a] | 179.89[a] | 3.5[a] | 100.28[a] |
| FI-1 | 3.67[a] | 55.11[a] | 5.33[a] | 182.45[a] | 4.5[b] | 124.21[b] |
| FI-2 | 3.64[a] | 68.89[b] | 5.56[b] | 210.87[b] | 5.0[b] | 129.29[b] |

*Note:* Comparisons were made among treatments for each parameter. Values with different symbols or letters are statistically different at $p < 0.05$.

**TABLE 7**
**Biological Parameters for Top Three Leaves in the CI, FI-1, and FI-2 Treatments Conducted at Guandong Ocean University Experiment Station in 2002**

| Treatment | Flag Leaf | | | Second Top Leaf | | | Third Top Leaf | | |
|---|---|---|---|---|---|---|---|---|---|
| | Length | Width | Angle | Length | Width | Angle | Length | Width | Angle |
| CI | 26.9[a] | 1.8[a] | 23.7[a] | 42.5[a] | 1.47[a] | 25.0[a] | 49.0[a] | 1.37[a] | 28.0[a] |
| FI-1 | 28.9[b] | 1.8[a] | 20.0[a] | 42[b] | 1.53[a] | 24.3[a] | 47.5[a] | 1.45[a] | 27.3[a] |
| FI-2 | 28.2[b] | 1.7[a] | 14.0[b] | 40.7[b] | 1.37[a] | 21.7[b] | 44.0[b] | 1.47[a] | 24.7[b] |

*Note*: Comparisons were made among treatments for each parameter. Values with different symbols or letters are statistically different at $p < 0.05$.

Dry weight of leaves for all treatments increased initially and then decreased over time. The dry weight for the CI was slightly higher than both treatments at the early growth stage, but became lower as the rice grew, with a significant difference between CI and FI at the final stage (Fig. 5). A similar pattern was also observed for the leaf sheath dry weight, but with the lowest dry weight for FI-2 (Fig. 6).

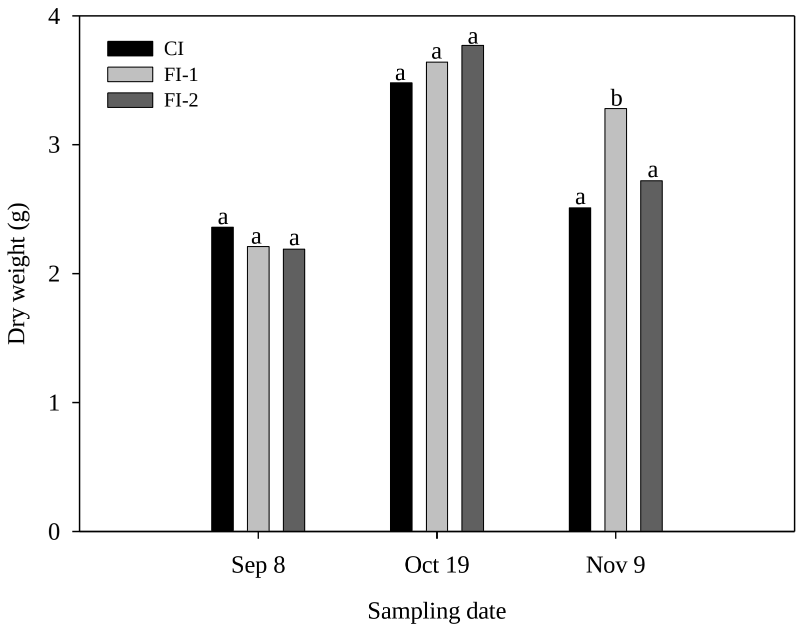

**FIGURE 5.** Comparisons of dry weight of leaf blade among treatments at different sampling dates. Different letters at the top of the bars indicate significant difference ($p < 0.05$).

Canopy chlorophyll content affects rice production. The total chlorophyll for the bottom second and third leaf was highest in FI-1, followed by FI-2 and CI (Fig. 7). There was a significant difference in canopy chlorophyll contents between FI and CI, but the difference between FI-1 and FI-2 was not significant. The difference in chlorophyll content for the second leaf among treatments was not significant. For the flag leaves, FI-2 was higher than FI-1, with the lowest in the CI**.**

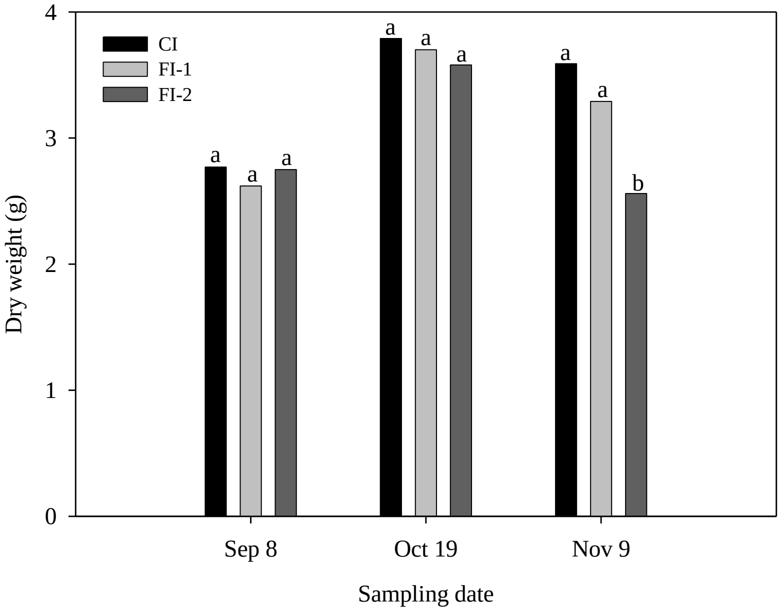

**FIGURE 6.** Comparisons of dry weight of leaf sheath among treatments at different sampling dates. Different letters at the top of the bars indicate significant difference ($p < 0.05$).

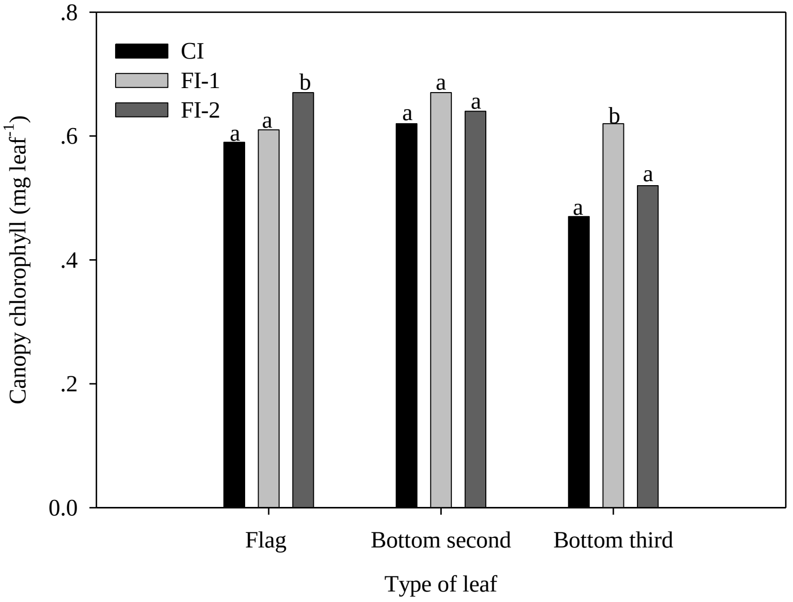

**FIGURE 7.** Comparisons of chlorophyll contents of canopy leaves among treatments. Different letters at the top of the bars indicate significant difference ($p < 0.05$).

The occurrence of rice sheath blight disease in different irrigation treatments was assessed during the 2003 experiment at Dongyang Research and Demonstration Station (Table 8). The infection rate averaged 44.4% for the CI system and 5.3% for the FI system.

Spikes in 0.25 $m^2$ were 96.7 for the CI system and 109.3 from the FI system, an increase of 13.05%. Other parameters in FI-1 were also higher than CI. Several parameters in FI-2 were lower than CI, resulting

**TABLE 8**
**Total and Infected Rice Plants in the CI and FI Treatments Conducted at Dongyang Research and Demonstration Station in the Leizhou Peninsula in Early 2003**

| Treatment | Replicate | Total | Infected | % Infected |
|---|---|---|---|---|
| CI | 1 | 300 | 124 | 41.3 |
| | 2 | 320 | 136 | 42.5 |
| | 3 | 300 | 148 | 49.3 |
| Average | | 307 | 136 | 44.4 |
| FI | 1 | 348 | 20 | 6.1 |
| | 2 | 332 | 16 | 4.9 |
| | 3 | 328 | 16 | 4.9 |
| Average | | 336 | 17.3 | 5.3 |

in lower production than FI-1, but still higher than that of the CI. For the 2003 early rice, the number of spikes averaged 295 for the CI, but 329 for the FI, which is an 11.53% increase over the former, indicating that the irrigation without water layer promoted tillering and the formation of spikes. Furthermore, the average grain weight per 1000 grains was 26.1 g for the CI and 27.1 g for FI (Table 9).

**TABLE 9**
**Grain Characteristics in the CI, FI-1, and FI-2 Treatments**

| Treatment | Effective Spikes (count) | Ear Length (cm) | Spikes (count) | Filled Spikes (count) | Setting Rate (%) | Wt/1000-Grains (g) |
|---|---|---|---|---|---|---|
| CI | $96.7^a$ | $20.7^a$ | $144.5^a$ | $130.7^a$ | $90.95^a$ | $24.1^a$ |
| FI-1 | $109.3^b$ | $21.4^a$ | $146.3^a$ | $132.1^a$ | $90.27^a$ | $24.4^a$ |
| FI-2 | $112.3^b$ | $20.5^a$ | $135.3^b$ | $123.2^b$ | $90.06^a$ | $24.2^a$ |

*Note*: Comparisons were made among treatments for each parameter. Values with different letters are statistically different at $p < 0.05$.

### DISCUSSION

In this study, I used the FI system with the width of the rice bed of 200 cm, and width and depth of the irrigation furrow of 20 cm and 15–20 cm, respectively, with no surface water layer overlying the rice bed except during the brief periods of seedling replanting and fertilization. These findings demonstrated that the FI technique met the water demand for growth, and also reduced water loss due to evaporation and seepage. Meanwhile, the FI technique also enhanced gas exchange in the soil, which provides a better growth environment for rice production. This technique is especially promising for areas of water shortage and also may serve as an effective means for water conservation in areas with competing water demands for agriculture, industry, and urban uses. For example, Bouman and Tuong[14] indicated that water saving under saturated soil was on average 23% with yield reduction of only 6%. Tao et al.[16] reported using a new water-saving Ground Cover Rice Production System (GCRPS) with lowland rice cultivated without a standing water layer during the entire growth period and plot irrigation when soil water tension was below 15 kPa. Compared to paddy control, only 32–54% of irrigation water was used in the GCRPS treatments, but yield in GCRPS with plastic cover was only 8% less than the paddy control yield in 2002. WUE in GCRPS was higher (0.35) than in paddy control (0.23). The experiment demonstrates that GCRPS has the potential to save substantial amounts of water with relatively minor yield penalties. Yang et al.[24] proposed the limiting values of soil water potential related to specific growth stages as irrigation indices. Compared with CI, the water-saving irrigation increased grain yield by 7.4 to 11.3%, reduced irrigation water by 24.5 to 29.2%, and increased water productivity by 43.1 to 50.3%. Nguyen et al.[12] indicated that water can be saved using a saturated soil culture system (SSC) with minimum effects on physiological processes by providing free water around 10 cm below the soil surface. However, Vories et al.[20] compared water savings and grain yields for the FI and flooded rice, and found that yields for flooded rice consistently exceeded that of the FI rice. On the other hand, Ockerby and Fukai[19] found that the grain yields of rice were slightly greater in paddy than on raised beds with continuous FI, but pointed out that all cultivars grown with the FI system had more tillers, leaf area, and dry weight at anthesis, suggesting a greater yield potential.

Studies on reduction of water use for rice farming have been widely conducted. However, the mechanisms controlling water loss have not been carefully assessed. For example, Tabbal et al.[9] indicated that intermittent irrigation in rice fields may reduce seepage. They found no difference in evapotranspiration between continuous flooding and intermittent irrigation. Tripathi et al.[25] found that intermittent irrigation reduced water seepage by 25 to 36% under different soil texture. They also believed that intermittent irrigation may reduce evapotranspiration. The average amount of evaporation/ evapotranspiration in the CI system is typically 7.4–9.2 mm day$^{-1}$, while intermittent irrigation averaged 6.1–7.1 mm day$^{-1}$. The pot simulation experiment conducted in the subtropical Leizhou Peninsula showed that compared to the CI, FI reduced water use by 38%. Reduction of evaporation, evapotranspiration, and seepage accounted for 25, 25, and 50% of the water savings, respectively. This finding is consistent with that reported by Tripathi et al.[25], who pointed to the major water-saving mechanism as the reduction in seepage, with the reduction of plant evaporation and evapotranspiration as the secondary means of water savings. Reduced seepage in the FI system is likely due to the low average water layer in the rice field and, hence, low seepage strength. The reduction of seepage under different soil texture, and hydrological, morphological, and geological conditions needs more studies.

This study revealed many advantages that led to lower water use and higher rice production in the FI over the CI system. FI reduced moisture content in the rice field, promoted light penetration and gas exchange, and lowered reduced materials, such as ferrous iron ($Fe^{2+}$) content in the soil, which in turn led to higher growth rate and rice production. This is reflected by the reduction in the population height and the number of tillers at the early stage of growth, the increases in the quality of tillers and the rate of spike formation, and the reduced occurrence of disease, such as the rice sheath blight. The improvements of rice growth under the FI system are also evident by higher dry mass weight of different plant components and the higher grain quality. Similarly, Lu et al.[8] found no difference in grain yield between a CI system and a reduced flooding irrigation. Bouman et al.[15] found that rice yield under aerobic conditions was 32–88% higher than under flooded conditions.

Lu et al.[8] found no difference in dry mass production between continuous flooding irrigation treatment and the reduced irrigation treatment. Ockerby and Fukai[19] found higher dry weight of all rice cultivars with FI than with flooding irrigation. This study showed that the dry weight of leaves, stems, roots, and sheaths in the direct sowing FI system increased initially and then decreased. The amount of root dry mass at all three growth stages was highest in FI-2, followed by FI-1 and CI, indicating that the root system from the FI system developed better than that from the flooding system. The stem dry mass at the early growth stage in the CI system was higher, but significantly lower than that from the FI system. This indicates that infiltration irrigation had better gas exchange and higher temperature, which facilitated root growth and longevity.

### CONCLUSIONS

WUE is an important measure of the relationship between water consumption and rice production. This study showed that rice production using the FI system was significantly higher than that of the CI system. The low irrigation system (FI-2) showed higher WUE than the high irrigation system (FI-1). Accompanied with a reduction of water use and the increases in rice production, my results also show improved soil condition and reduced rice disease. Future study should focus on FI systems that maximize rice production and minimize water consumption.
